# Very old patients admitted to intensive care in Australia and New Zealand: a multi-centre cohort analysis

**DOI:** 10.1186/cc7768

**Published:** 2009-04-01

**Authors:** Sean M Bagshaw, Steve AR Webb, Anthony Delaney, Carol George, David Pilcher, Graeme K Hart, Rinaldo Bellomo

**Affiliations:** 1 Department of Intensive Care, Austin Hospital, Studley Road, Heidelberg, VIC 3084, Australia; 2 Division of Critical Care Medicine, University of Alberta Hospital, University of Alberta, Walter C Mackenzie Centre, 8440-112 ST NW, Edmonton, Alberta T6G 2B7, Canada; 3 Department of Intensive Care, Royal Perth Hospital, Wellington Street, Perth, WA 6000 Australia; 4 School of Population Health, University of Western Australia, Crawly, Perth, WA 6009, Australia; 5 Intensive Therapy Unit, Royal North Shore Hospital, and Northern Clinical School, University of Sydney, St Leonards, Sydney, NSW 2065, Australia; 6 Australia New Zealand Intensive Care Society (ANZICS) Clinical Outcomes and Resource Evaluation Centre, Carlton, 10 Ievers Terrace, VIC 3053, Australia; 7 Department of Intensive Care Medicine, Alfred Hospital, Commercial Road, Prahran, VIC 3181, Australia; 8 Australian and New Zealand Intensive Care Research Centre, Department of Epidemiology and Preventive Medicine, Monash University, Melbourne, VIC 3004 Australia

## Abstract

**Introduction:**

Older age is associated with higher prevalence of chronic illness and functional impairment, contributing to an increased rate of hospitalization and admission to intensive care. The primary objective was to evaluate the rate, characteristics and outcomes of very old (age ≥ 80 years) patients admitted to intensive care units (ICUs).

**Methods:**

Retrospective analysis of prospectively collected data from the Australian New Zealand Intensive Care Society Adult Patient Database. Data were obtained for 120,123 adult admissions for ≥ 24 hours across 57 ICUs from 1 January 2000 to 31 December 2005.

**Results:**

A total of 15,640 very old patients (13.0%) were admitted during the study. These patients were more likely to be from a chronic care facility, had greater co-morbid illness, greater illness severity, and were less likely to receive mechanical ventilation. Crude ICU and hospital mortalities were higher (ICU: 12% vs. 8.2%, *P *< 0.001; hospital: 24.0% vs. 13%, *P *< 0.001). By multivariable analysis, age ≥ 80 years was associated with higher ICU and hospital death compared with younger age strata (ICU: odds ratio (OR) = 2.7, 95% confidence interval (CI) = 2.4 to 3.0; hospital: OR = 5.4, 95% CI = 4.9 to 5.9). Factors associated with lower survival included admission from a chronic care facility, co-morbid illness, nonsurgical admission, greater illness severity, mechanical ventilation, and longer stay in the ICU. Those aged ≥ 80 years were more likely to be discharged to rehabilitation/long-term care (12.3% vs. 4.9%, OR = 2.7, 95% CI = 2.6 to 2.9). The admission rates of very old patients increased by 5.6% per year. This potentially translates to a 72.4% increase in demand for ICU bed-days by 2015.

**Conclusions:**

The proportion of patients aged ≥ 80 years admitted to intensive care in Australia and New Zealand is rapidly increasing. Although these patients have more co-morbid illness, are less likely to be discharged home, and have a greater mortality than younger patients, approximately 80% survive to hospital discharge. These data also imply a potential major increase in demand for ICU bed-days for very old patients within a decade.

## Introduction

The global population is aging. This trend results from a process referred to as demographic transition, characterized by declines in both fertility and mortality rates [1]. The probability of survival to older age has improved and the absolute number and proportion of older persons is projected to increase in the next few decades [1]. The fastest growing age cohort is made up of those aged ≥ 80 years, increasing at an estimated 3.8% per year and projected to represent one-fifth of all older persons by 2050 [1].

Older age is associated with an increased prevalence of chronic illness and functional impairment [2,3]. As a result, the rate of hospitalizations for acute illness among older persons is certain to increase [4]. Similarly, the demand for critical care services and admissions to intensive care units (ICUs) is also projected to dramatically rise in the next decade [5]. Data from the United States estimates approximately 55% of all ICU bed-days are incurred by patients aged ≥ 65 years and an estimated 14% of those patients aged ≥ 85 years die in the ICU [5]. There are conflicting data, however, on the short-term and long-term survival for older patients admitted to the ICU [6-15]. These disparities may reflect differences in the severity and type of illness, length of follow-up, definitions for old age, and treatment intensity for older patients [12,16,17].

Owing to the aging population, an evaluation of how best to provide care for acutely ill older patients and to optimize recovery has become an important issue that may have implications on health resources in terms of triage, decision-making, expansion of ICU capacity, and advanced care planning. Moreover, there is an urgent need to understand the implications on outcomes for older patients after ICU admission, including not only survival but also cognitive impairment, quality-of-life, and functional autonomy [18-23].

Accordingly, we interrogated the Australian and New Zealand Intensive Care Society Clinical Outcomes and Resource Evaluation (ANZICS CORE) Adult Patient Database (APD) to obtain information on very old patients (age ≥ 80 years) from 57 Australian hospitals over a 6-year period. Our primary objectives were to evaluate the cumulative (and annual) change in the proportion of very old patients admitted to the ICU, to evaluate the clinical characteristics and the cumulative (and 6-year trends) outcomes of very old patients compared with those aged < 80 years, to evaluate factors associated with survival for very old patients admitted to the ICU, and to project estimates of ICU admission rates and of ICU and hospital bed-days for this cohort.

## Materials and methods

### Study population and setting

The present study was a retrospective analysis of prospectively collected data. We interrogated the ANZICS CORE APD for all ICU admissions for ≥ 24 hours from 1 January 2000 to 31 December 2005. The ANZICS CORE APD is a clinical database containing data from > 700,000 individual adult admissions to 183 ICUs from 1987 to the present, and captures nearly 70% of all ICU admissions in Australia and New Zealand (ANZ). These data provide a realistic representative sampling of all ICU admissions in ANZ [24]. In the event of multiple admissions, only the initial ICU admission was considered. Those patients re-admitted within 72 hours after initial discharge were considered part of the index admission. We selected ICUs that had continuously contributed data to the APD during this 6-year period. The sample comprised 57 ICUs (19 tertiary referral hospitals, 15 metropolitan hospitals, 12 regional/rural hospitals and 11 private hospitals).

Access to the data was granted by the ANZICS CORE Management Committee in accordance with standing protocols. Data are collected primarily for ICU outcome peer review under the Quality Assurance Legislation of the Commonwealth of Australia (Part VC Health Insurance Act 1973, Commonwealth of Australia). Such data are collected and transferred from hospitals to the database with government support and funding. Hospital data are submitted by or on behalf of the ICU Director and results are reported back to the Director. Each hospital allows subsequent data use as appropriate under the ANZICS CORE standing procedures and in compliance with the ANZICS CORE Terms of Reference [25].

### Data collection

Standard demographic, clinical, and physiologic data were retrieved. Demographic information included age, sex, dates and source of admission, and dates and disposition at hospital discharge. Clinical data encompassed the primary diagnosis, the surgical status (that is, emergency surgery, cardiac surgery, trauma-related surgery), the presence of co-morbidities, and the need for mechanical ventilation. Physiologic data included the urine output and laboratory data. Severity of illness was assessed using the Acute Physiology and Chronic Health Evaluation (APACHE) II and APACHE III scoring systems [26]. The definitions regarding pre-existing co-morbidities, primary diagnostic categories, and acute kidney injury are presented in Additional data file 1.

### Outcome measures

The primary outcome – the proportion of total admissions of patients aged ≥ 80 years – was described as a proportion annually and cumulatively. These data were compared with the admission rates for age strata of 18 to 40 years, 40.1 to 64.9 years, and 65 to 79.9 years, respectively.

To estimate whether a change in the proportion of admissions of patients aged ≥ 80 years occurred over the study period, a straight-line regression of the natural logarithm of the proportion of admissions aged ≥ 80 years was fitted with calendar year as the independent variable. The estimated annual percentage change was equal to [100 × (exp(*b*) – 1)], where *b *represents the slope of the regression. If the estimated annual percentage change is statistically greater than zero, then the proportion of admissions of patients aged ≥ 80 years had an increasing trend over the study period [27].

Crude and adjusted ICU and hospital mortality rates for those patients aged ≥ 80 years were compared with other age strata. Clinical factors associated with hospital survival for those patients aged ≥ 80 years were evaluated. Subgroup analyses were also performed for those patients aged ≥ 85 and ≥ 90 years, respectively.

### Statistical analysis

Analysis was performed using Intercooled Stata Release 10 (Stata Corp, College Station, TX, USA). In the event of missing data values, data were not replaced. Normally distributed or near-normally distributed variables are reported as means with standard deviations and were compared by Student's *t *test, analysis of variance, or simple linear regression. Non-normally distributed continuous data are reported as medians with interquartile ranges and were compared by the Mann–Whitney U test or the Kruskal–Wallis test. Categorical data were reported as proportions and were compared using Fisher's exact test.

Multivariable logistic regression analysis was used to account for potential confounding variables in the association of age strata and the ICU and hospital mortalities. The admission source, sex, co-morbid disease, surgical status, primary diagnosis, need for mechanical ventilation, nonage-related APACHE II score (subtraction of age-related points from the full APACHE II score [28]), and hospital site were *a priori *covariates for this analysis.

A second multivariable logistic regression analysis was used to evaluate for factors associated with hospital survival for the cohort aged ≥ 80 years. Covariates initially considered for this analysis included the admission source, sex, co-morbid disease, surgical status, primary diagnosis, need for mechanical ventilation, nonage-related APACHE II score, duration of ICU stay, and hospital site.

Model fit was assessed by the goodness-of-fit test, and discrimination was assessed by the area under the receiver operator characteristic curve. Data are presented as odds ratios (ORs) with 95% confidence intervals (CIs). Standardized mortality ratios were calculated by the ratio of observed inhospital death to predicted inhospital mortality by the APACHE II score. Sex-specific incidence rate ratios (95% CI) stratified by age category were calculated to compare admission rates. Sensitivity analysis was performed based on calculated annual admission rates for patients aged ≥ 80 years and was extrapolated for all of ANZ to project the estimated resource demand through 2015. *P *< 0.05 was considered statistically significant for all comparisons.

## Results

**Figure 1:**
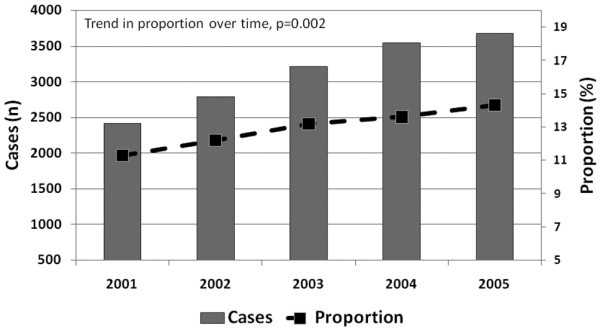
Intensive care unit admissions for patients aged ≥ 80 years. Absolute number and proportion of intensive care unit admissions for patients aged ≥ 80 years from the Australian and New Zealand Intensive Care Society Adult Patient Database 2001 to 2005.

During the 6-year study period, 124,088 patients were admitted to the 57 ICUs, and 120,123 (96.8%) patients had adequate data for evaluation. The cumulative proportion of patients aged ≥ 80 years admitted during the study period was 13.0% (n = 15,640). The absolute number and the proportion of patients aged ≥ 80 years admitted to the ICU significantly increased annually (Figure 1). There was an estimated 5.6% annual increase (95% CI = 3.8% to 7.3%, *P *= 0.002) in patients aged ≥ 80 years admitted during the study period.

### Patient characteristics

A summary of patient characteristics, admission details, primary diagnoses, and acute physiology is presented in Tables 1 and 2. Further stratification by age decile is shown in Additional data file 2. Males had a higher rate of ICU admission across all strata of age categories when compared with females. This association was more pronounced for age strata ≥ 50 years (see Additional data file 3).

**Table 1:** Summary of patient demographics, admission details and primary diagnoses by age strata

Characteristics	Total (n = 120,123)	Age strata				*P *value
			
		18 to 40 years (n = 16,732)	40.1 to 64.9 years (n = 42,285)	65 to 79.9 years (n = 45,466)	≥ 80 years (n = 15,640)	
Age (years)	61.7 (17.5)	29.4 (6.5)	54.4 (7.0)	72.7 (4.2)	84.2 (3.5)	<0.0001
Male sex	59.5	57.0	61.5	61.4	51.1	<0.0001
Hospital admission source						
Home	79.2	74.4	79.0	81.0	79.8	<0.001
Other acute care hospital	17.2	22.3	18.3	16.3	15.6	
Chronic care facility	1.3	0.6	0.8	1.2	3.3	
Other intensive care unit	1.8	2.6	1.9	1.6	1.3	
Co-morbid disease						
Any	28.6	11.0	28.3	34.1	32.1	<0.001
≥ 2	6.5	2.5	6.3	7.8	7.3	<0.001
Specific co-morbid diseases						
Cardiovascular	15.6	2.3	12.0	21.1	23.5	<0.001
Respiratory	8.4	3.2	7.6	10.7	9.5	<0.001
Immunocompromised	4.9	3.8	6.1	4.8	3.2	<0.001
Metastatic cancer	2.9	1.0	3.5	3.3	2.3	<0.001
Hepatic	2.3	2.3	4.1	1.2	0.5	<0.001
End-stage kidney disease	3.4	1.5	3.1	4.0	4.1	<0.001
Haematologic malignancy	1.7	1.3	2.1	1.7	1.1	<0.001
Admission details						
Nonelective admission	61.0	83.4	60.5	53.0	61.8	<0.001
Surgical admission	49.7	29.8	48.6	56.8	53.0	<0.001
Cardiovascular	46.1	13.5	44.5	55.6	39.5	<0.001
Trauma	7.9	24.9	6.7	3.4	5.6	<0.001
Emergency surgical	31.3	62.5	28.6	25.3	38.1	<0.001
Primary diagnosis						
Sepsis/septic shock	27.8	28.7	28.4	27.0	27.5	<0.001
Respiratory	11.7	12.0	12.5	11.4	10.0	<0.001
Neurologic	9.3	13.3	12.2	6.6	5.1	<0.001
Cardiac	9.3	4.7	8.6	10.5	12.3	<0.001
Gastrointestinal (other)	8.8	2.5	7.2	10.4	15.0	<0.001
Hepatic	5.9	4.0	6.5	5.5	7.4	<0.001
Metabolic/poisoning	5.3	16.8	5.9	1.7	1.7	<0.001
Gastrointestinal bleeding	2.3	1.3	2.3	2.2	4.0	<0.001

Data presented as mean (standard deviation) or percentage.

**Table 2:** Summary of illness severity and selected laboratory values by age strata

Characteristic	Total (n = 120,123)	Age strata				*P *value
			
		18 to 40 years (n = 16,732)	40.1 to 64.9 years (n = 42,285)	65 to 79.9 years (n = 45,466)	≥ 80 years (n = 15,640)	
Illness severity scores						
APACHE II	16.9 (7.7)	13.0 (7.4)	15.3 (7.6)	18.7 (7.2)	19.8 (7.1)	<0.001
Nonage-related APACHE^a^	13.3 (7.3)	13.1 (7.4)	13.1 (7.6)	13.4 (7.2)	13.8 (7.1)	<0.001
APACHE III	55.1 (27.5)	42.3 (26.8)	49.4 (27.2)	60.8 (25.6)	67.5 (25.0)	<0.001
Mechanical ventilation (%)	52.0	52.9	53.7	53.1	43.7	<0.001
Creatinine (μmol/l)	90 (68 to 130)	75 (56 to 98)	80 (61 to 111)	98 (71 to 141)	110 (80 to 160)	<0.001
Urea (mmol/l)	6.6 (4.6 to 10.8)	4.5 (3.2 to 6.4)	5.9 (4.2 to 9.0)	7.6 (5.4 to 12)	9.4 (6.5 to 14.7)	<0.001
Urine output (l/24 hours)	1.9 (1.3 to 2.7)	2.3 (1.5 to 3.4)	2.0 (1.3 to 2.9)	1.8 (1.2 to 2.6)	1.6 (1.0 to 2.3)	<0.001
Acute kidney injury (%)	36.1	17.7	27.4	44.1	56.4	<0.001

Data presented as mean (standard deviation), percentage, or median (intraquartile range). SI conversion rates: serum creatinine, 1 mg/dl = 88.4 μmol/l; serum urea, 1 mg/dl = 0.357 mmol/l. ^a^Acute Physiology and Chronic Health Evaluation (APACHE) II score minus points for age.

Patients aged ≥ 80 years were more likely to be admitted from a chronic care facility (OR = 3.66, 95% CI = 3.3 to 4.1, *P *< 0.001). The prevalence of more than one co-morbid illness was significantly higher for patients aged ≥ 65 years (*P *< 0.0001 for each); however, there was no clinically important difference between patients aged 65 to 79.9 years and patients aged ≥ 80 years (34.1% vs. 32.2%, respectively). Patients aged ≥ 80 years had comparable rates of sepsis but lower rates of neurologic and metabolic-related diagnoses and higher rates for cardiac and gastrointestinal-related admission compared with younger age strata. Patients aged ≥ 80 years had greater severity of illness (nonage-related APACHE II score, 13.8 for patients aged ≥ 80 years vs. 13.2 for patients aged < 80 years, *P *< 0.0001) and higher rates of acute kidney injury (OR = 2.6, 95% CI = 2.5 to 2.7, *P *< 0.0001), but fewer received mechanical ventilation (OR = 0.68, 95% CI = 0.66 to 0.70, *P *< 0.0001).

### Survival

**Figure 2:**
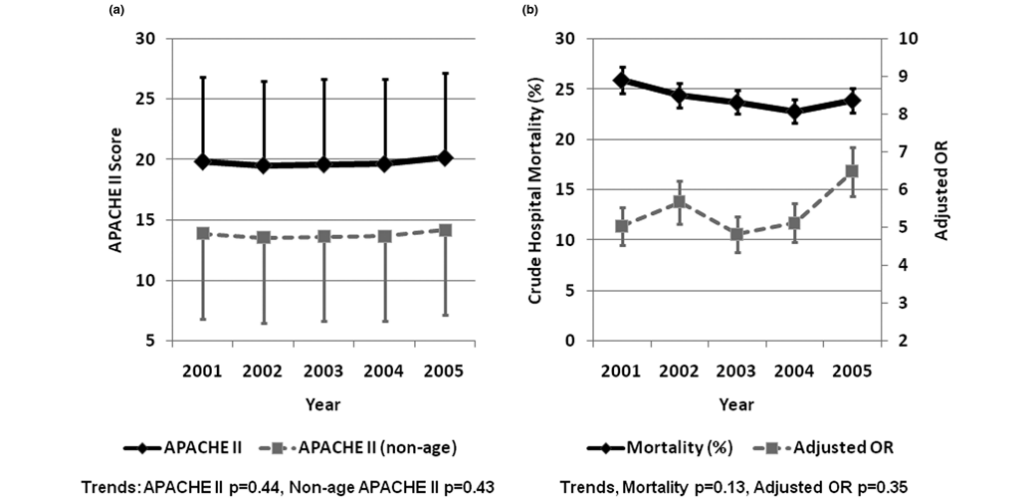
Severity of illness and outcomes for patients aged ≥ 80 years. Trends in severity of illness and outcomes for patients aged ≥ 80 years from the Australian and New Zealand Intensive Care Society Adult Patient Database 2001 to 2005. **(a) **Mean and standard deviation Acute Physiology and Chronic Health Evaluation (APACHE) II and nonage APACHE II scores. **(b) **Crude mortality with 95% confidence interval and adjusted odds ratio (OR) with 95% confidence interval for death.

Trends in the severity of illness, crude mortality, and adjusted OR for death are shown in Figure 2. The cumulative crude and adjusted ICU and hospital mortalities were significantly higher for patients aged ≥ 80 years when compared with all other age strata (Table 3). This cohort also had a higher standardized mortality ratio (1.28, 95% CI = 1.19 to 1.36) when compared with younger age strata (see Additional data file 2).

**Table 3:** Summary of predicted, crude and adjusted intensive care unit and hospital mortalities

Age strata	Crude mortality (%)		Predicted mortality (%)		ICU mortality (odds ratio (95% confidence interval))		Hospital mortality (odds ratio (95% confidence interval))	
	
	ICU	Hospital	APACHE II	APACHE III	Crude	Adjusted^a^	Crude	Adjusted^b^
18 to 40 years^c^	5.6	7.1	14.6	10.1	1.0	1.0	1.0	1.0
40.1 to 64.9 years	7.6	11.4	22.5	15.3	1.39 (1.3 to 1.5)	1.44 (1.3 to 1.6)	1.69 (1.6 to 1.8)	1.77 (1.6 to 1.9)
65 to 79.9 years	9.8	16.6	30.1	21.7	1.85 (1.7 to 2.0)	2.13 (1.9 to 2.3)	2.62 (2.5 to 2.8)	3.17 (2.9 to 3.4)
≥ 80 years	12.0	24.0	32.7	25.3	2.30 (2.1 to 2.5)	2.70 (2.4 to 3.0)	4.16 (3.9 to 4.5)	5.37 (4.9 to 5.9)

APACHE, Acute Physiology and Chronic Health Evaluation; ICU, intensive care unit. ^a^Goodness of fit, *P *= 1.0; area under the receiver operator characteristic curve = 0.87. ^b^Goodness of fit, *P *= 1.0; area under the receiver operator characteristic curve = 0.85. ^c^Reference variable.

**Figure 3:**
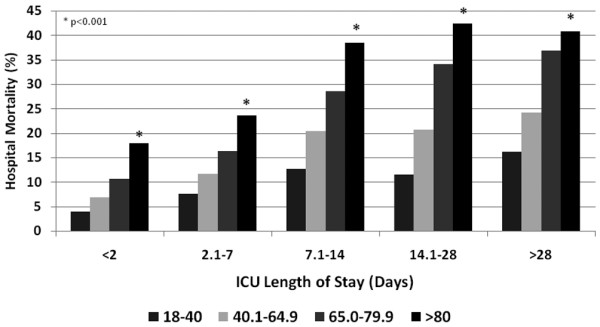
Hospital mortality and intensive care unit (ICU) length of stay by age category from the Australian and New Zealand Intensive Care Society Adult Patient Database 2001 to 2005.

Several factors were independently associated with higher odds of death for patients aged ≥ 80 years in multivariable analysis (Table 4). Admission from a chronic care facility was associated with a significantly lower survival to hospital discharge (75.5% vs. 85.8%, *P *< 0.001). Those patients with co-morbid illness, a nonsurgical admission, higher acuity of illness, need for mechanical ventilation, and evidence of acute kidney injury had lower survival. A longer duration of stay in the ICU was also associated with lower hospital survival (Figure 3).

**Table 4:** Summary of factors associated with hospital survival for patients aged ≥ 80 years

Factor	Odds ratio (95% confidence interval)	*P *value
Admission from chronic care facility	1.35 (1.09 to 1.67)	0.005
Co-morbid disease (present)		
≤ 1	1.0^a^	
≥ 2	1.31 (1.12 to 1.52)	0.001
Admission type (present)		
Elective surgical	1.0^a^	
Emergency surgical	1.83 (1.58 to 2.13)	<0.001
Medical	2.58 (2.22 to 3.00)	<0.001
Admission diagnosis (present)		
Sepsis	1.24 (1.10 to 1.40)	<0.001
Trauma	1.28 (1.05 to 1.57)	0.016
Hepatic	1.21 (1.02 to 1.44)	0.025
Gastrointestinal (nonbleeding)	1.72 (1.48 to 1.99)	<0.001
Cardiac	1.54 (1.34 to 1.77)	<0.001
Neurologic	1.92 (1.59 to 2.33)	<0.001
Respiratory	1.29 (1.11 to 1.49)	0.01
Metabolic	0.53 (0.36 to 0.76)	0.01
Nonage-related APACHE II score (per point)	1.11 (1.10 to 1.11)	<0.001
Mechanical ventilation (present)	1.18 (1.07 to 1.30)	0.001
Acute kidney injury (present)	1.38 (1.25 to 1.51)	<0.001
ICU length of stay (log-transformed) (per day)	1.17 (1.11 to 1.24)	<0.001

Model also included adjustment for hospital site. Goodness of fit, *P *= 1.0; area under the receiver operator characteristic curve = 0.79. APACHE, Acute Physiology and Chronic Health Evaluation; ICU, intensive care unit. ^a^Reference variable.

### Secondary outcomes

**Figure 4:**
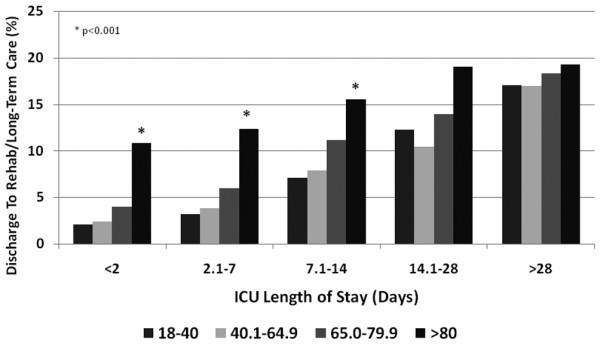
Discharge to rehabilitation or long-term care facility and intensive care unit (ICU) length of stay by age category from the Australian and New Zealand Intensive Care Society Adult Patient Database 2001 to 2005.

The ICU length of stay was shorter for those patients aged ≥ 80 years not surviving; however, it was greater for survivors when compared with other age strata (Table 5). For both survivors and nonsurvivors, the total duration of hospitalization was longer for patients aged ≥ 80 years. While a majority of patients aged ≥ 80 years was discharged home from hospital, this cohort was also more likely to be discharged from hospital to a rehabilitation/long-term care facility (12.3% vs. 4.9%; OR = 2.7, 95% CI = 2.6 to 2.9, *P *< 0.0001). Admission to hospital from a chronic care facility was significantly predictive of discharge to a rehabilitation/long-term care facility (33.9% vs. 11.5%; OR = 3.9, 95% CI = 3.1 to 5.0, *P *< 0.0001). Higher acuity of illness (nonage-related APACHE II score, 12.8 vs. 12.1; *P *= 0.0001) and longer duration of stay in the ICU were also associated with a greater likelihood of discharge to a rehabilitation/long-term care facility (Figure 4).

**Table 5:** Summary of secondary clinical outcomes

Clinical outcome	Total (n = 120,123)	Age strata				*P *value
			
		18 to 40 years (n = 16,732)	40.1 to 64.9 years (n = 42,285)	65 to 79.9 years (n = 45,466)	≥ 80 years (n = 15,640)	
ICU length of stay (days)						
Dead	3.9 (2.0 to 8.7)	4.4 (2.1 to 9.0)	4.0 (2.0 to 8.7)	3.9 (2.0 to 8.6)	3.5 (1.9 to 7.0)	0.0003
Alive	2.5 (1.7 to 4.8)	2.4 (1.6 to 4.9)	2.3 (1.6 to 4.6)	2.3 (1.7 to 4.3)	2.6 (1.7 to 4.5)	0.0001
Hospital length of stay (days)						
Dead	9.7 (4.0 to 21.6)	6.7 (2.9 to 17.3)	9.0 (3.7 to 20.9)	10.3 (4.2 to 22.9)	10.0 (4.5 to 20.7)	0.0001
Alive	11.8 (7.1 to 21.8)	9.0 (4.6 to 19.3)	10.9 (6.9 to 20.6)	12.7 (8.0 to 22.0)	14.9 (9.1 to 25.8)	0.0001
Discharge location of survivors (%)						
Home	83.2	84.9	86.0	83.1	72.2	
Transfer to other hospital	11.1	11.2	9.9	10.9	15.1	<0.001
Rehabilitation/long-term care	5.7	3.8	4.1	6.1	12.3	

Data presented as median (interquartile range) or percentage. ICU, intensive care unit.

### Subgroup of ICU admissions in patients aged ≥ 85 years

The cumulative proportion admitted to the ICU for patients aged ≥ 85 years was 4.2% (n = 5,049). The annual rate increased significantly over the study period by 18.5% (95% CI = 9.5 to 27.4, *P *= 0.007). The mean (standard deviation) APACHE II and nonage-related APACHE II scores were 19.8 (7.0) and 13.8 (7.0), with a nonsignificant trend over the study period (*P *= 0.08). Cumulative ICU and hospital mortalities were 12.8% and 27.6%, respectively. There was a reduction in crude hospital mortality (-20%; 95% CI to -31 to -9, *P *= 0.009); however, there was no change in the adjusted OR for death over the study period.

### Subgroup of ICU admissions in patients aged ≥ 90 years

The cumulative proportion admitted to the ICU for patients aged ≥ 90 years was 0.88% (n = 1,056). There was a similar annual increase in the admission rate over the study of 6.6% (95% CI = 3.6% to 15.69%, *P *= 0.02). The mean (standard deviation) APACHE II and nonage-related APACHE II scores were 19.8 (7.0) and 13.8 (7.0), with no significant trends over the study period (*P *= 0.66). The cumulative ICU and hospital mortalities were 12.0% and 26.7%, respectively. There were no trends in either crude OR (*P *= 0.08) or adjusted OR (*P *= 0.37) for death. A comparison of crude and adjusted ICU and hospital mortalities for subgroups aged ≥ 80 years is presented in Table 6.

**Table 6:** Summary of crude and adjusted odds ratios of death by age strata ≥ 80 years

Age strata	Crude mortality (%)		ICU mortality (odds ratio (95% confidence interval))		Hospital mortality (odds ratio (95% confidence interval))	
	
	ICU	Hospital	Crude	Adjusted^a^	Crude	Adjusted^b^
80 to 84.9 years	11.6	22.4	1.0^c^	1.0^c^	1.0^c^	1.0^c^
85 to 89.9 years	13.0	27.0	1.14 (1.02 to 1.27)	1.19 (1.04 to 1.36)	1.28 (1.18 to 1.40)	1.32 (1.20 to 1.46)
≥ 90 years	11.9	29.6	1.03 (0.85 to 1.25)	1.16 (0.93 to 1.46)	1.46 (1.27 to 1.68)	1.71 (1.46 to 2.01)

ICU, intensive care unit. ^a^Goodness of fit, *P *= 1.0; area under the receiver operator characteristic curve = 0.82. ^b^Goodness of fit, *P *= 1.0; area under the receiver operator characteristic curve = 0.80. ^c^Reference variable.

### Sensitivity analysis and resource projection

**Figure 5:**
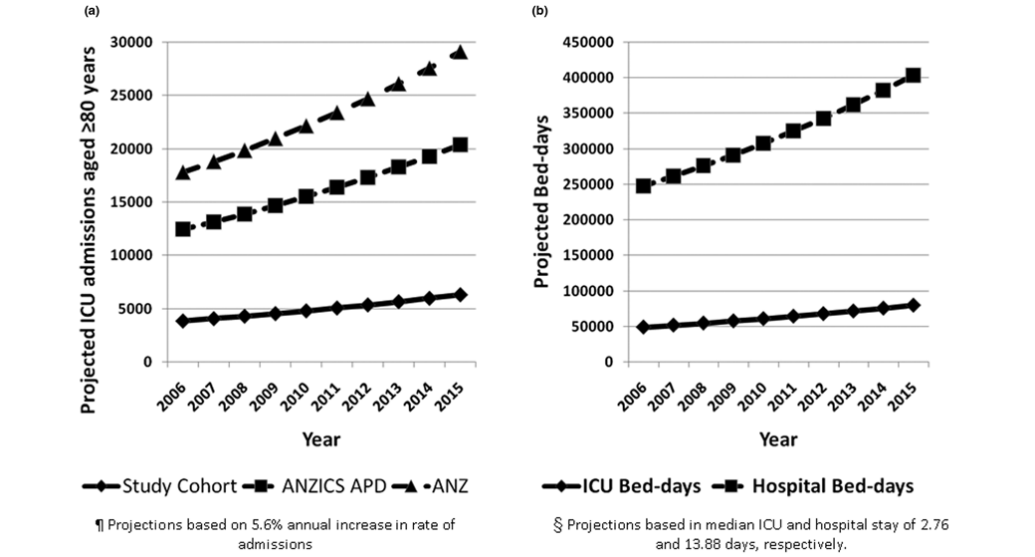
Projected intensive care unit and hospital estimations for patients aged ≥ 80 years. **(a) **Projected intensive care unit (ICU) admissions and **(b) **projected ICU and hospital bed-days for patients aged ≥ 80 years for Australia and New Zealand (ANZ) from 2006 to 2015. ANZICS APD, Australian and New Zealand Intensive Care Society Adult Patient Database.

Estimations of the projected increase in both ICU admissions and ICU and hospital bed-days for patients aged ≥ 80 years are shown in Figure 5. This sensitivity analysis assumes a linear 5.6% annual increase in admission rates and shows the potential projected resource utilization for patients aged ≥ 80 years through to 2015. These data indicate the potential for a 72.4% increase in ICU and hospital bed-days for patients aged ≥ 80 years by 2015 when compared with 2005.

## Discussion

We performed a 6-year retrospective analysis of over 120,000 ICU admissions to 57 ICUs across ANZ, using a large validated clinical database, to evaluate the rate, clinical characteristics, outcomes and projected resource demand of very old patients (aged ≥ 80 years) admitted to the ICU.

Our study found that very old patients represented 13.0% of all patients admitted to the ICU and this rate increased by an estimated 5.6% annually during the study period. We found similar increases in the annual admission rates for patients aged ≥ 85 and ≥ 90 years. Interestingly, we showed evidence of sex-specific differences in ICU admission rates, with males higher than females, and this was modified by age, with by greater differences in older age strata. We also found that very old patients were more likely to be admitted from chronic care facilities and to have a higher burden of co-morbid illnesses. Similarly, very old patients presented with greater acuity of illness (after accounting for the age points in APACHE II score) yet were less likely to receive mechanical ventilation while in the ICU. This cohort also showed interesting differences in acute physiology and laboratory parameters, including higher serum creatinine, lower urine output, and greater occurrence of early acute kidney injury. Very old patients, when compared with younger age strata, showed consistently lower crude and adjusted ICU and hospital survival. Moreover, these patients were more likely to be admitted from chronic care facilities, have longer durations of stay in the ICU and in the hospital, and were significantly more likely to be discharged to a rehabilitation/long-term care facility.

Importantly, our findings have potential implications for future health resource demand, utilization and planning. These data imply the potential for a 72.4% projected increase in demand for ICU and hospital bed-days for very old patients within a decade.

Survival to older age has improved and contributed to the evolving demographic transition. Current global growth rates of persons aged ≥ 80 years (3.8% per year) are greater than any younger segment of the older population and are twice the growth rate of persons aged ≥ 60 years (1.9%) [1]. This trend, at present, is largely dominated by growth in more developed countries [1]. This projected increase will have importance on the demand and delivery of health services; in particular, intensive care.

Prior data have estimated ICU admission rates in the range 3.0% to 16.5% for patients aged ≥ 80 years [9,13,15,20,29]. These apparent differences are probably attributed to variations in study design, cultural/geographic variations and differences in the study populations (that is, severity of illness, definitions for old age, treatment intensity/triage). In comparison with these studies, the proportion of ICU admissions for very old patients in ANZ is relatively high. In addition, our large observational data have clearly shown an increasing trend in admission rates for this cohort. Moreover, this trend would be anticipated to continue and/or accelerate, and would certainly be expected to impact on health resources. For example, assuming a stable estimated average cost per patient ICU bed-day for nonchargeable patients in New South Wales (2004/2005) of AUS$3,999 [30] and a stable median ICU length of stay (that is, 2.76 days), the estimated increment in resource expenditures for ANZ (population~25 million) due to ICU bed-days for very old patients alone would be AUS$134 million per year by 2015 (AUS$681 million cumulative). This estimation, however, does not address the projected need for increased capacity.

Several investigations have shown that very old patients admitted to the ICU receive less aggressive treatment, including mechanical ventilation, when compared with younger cohorts, despite comparable illness severity scores [13,15,16]. Boumendil and colleagues conducted a matched-cohort study of over 6,000 patients comparing those aged 65 to 79 years and those aged ≥ 80 years [16]. Patients were matched by sex, surgical status, co-morbid disease, and illness severity. They found that fewer very old patients received mechanical ventilation or renal replacement therapy [16]. Hamel and colleagues found reduced resource intensity for older patients and a greater likelihood of having renal replacement therapy, vasopressors, tube feeding, and major surgical interventions withheld compared with younger patients [31]. Likewise, there were similar findings in our data – with very old patients less likely to receive mechanical ventilation.

Whether these differences represent active or passive therapeutic limits recommended by clinicians, the preferences by families or patients, or the result of unaccounted confounding factors remains uncertain. This may, however, also indicate that there is a sizeable selection bias for which very old patients are triaged access to ICU support [12,16]. During a 20-month prospective, single-centre study of 180 very old patients triaged for the ICU, Garrouste-Orgeas and colleagues found that 73% were refused admission [12]. The explanations cited included being too well (28%) or too sick (44%) to benefit from ICU support. Further, Boumendil and colleagues found that those very old patients admitted were in reasonably good health, had fewer fatal underlying diseases, and had fewer complicated surgical interventions. This was also supported by the lower total costs of ICU admission for very old patients, largely attributable to a shorter ICU stay and less invasive therapies (that is, mechanical ventilation, tracheostomy, renal replacement therapy) [16,32]. These data potentially imply that a comprehensive assessment and careful selection of very old patients for ICU support may deliver positive short-term clinical outcomes.

Similar to prior observational studies, we found evidence of sex-specific differences in admission rates to the ICU [33-35]. Moreover, this difference was notably modified by increasing age, with males consistently more likely than females to be admitted to the ICU. In a retrospective Canadian analysis of 24,778 consecutive adult ICU admissions over a 2-year period, Fowler and colleagues found significant age-specific and sex-specific differences in ICU admission rates [33]. While a greater number of older females (aged ≥ 50 years) were admitted to hospital, older males were more likely – after covariate adjustment for the diagnostic category and illness severity – to be admitted to the ICU and to receive other ICU-related interventions. Older females also had shorter ICU durations of stay and higher adjusted ICU and hospital mortality. Likewise, in a prospective European of 25,998 consecutive ICU admissions to 31 centres over 3 years, Valentin and colleagues showed similar disparities in sex-specific admission rates to the ICU and in ICU-related interventions; however, there were no differences in survival [35].

Whether these findings represent genuine age-related and/or sex-related differences in access to the ICU and related interventions, represent variation in patient preferences for provision of ICU support, represent other health policy or decision-making processes, or were unaccounted for confounding remains uncertain [33]. This was a secondary finding of our study, and as such has not been specifically examined in detail. Owing to the consistency of these data across diverse health jurisdictions, however, further prospective evaluation is warranted.

Substantial effort has been dedicated to determining whether age is a primary determinant of ICU outcome for very old patients [6-9,11,13-15,20,29,36-39]. In our cohort, when compared with younger subgroups, older patients had greater odds of death in the ICU and the hospital after covariate adjustment that included co-morbid disease and severity of illness. Hamel and colleagues found that age was independently associated with lower short-term survival in older patients admitted to the ICU that was not attributable to older patients receiving less intensive therapy [13]. Moreover, patients aged ≥ 80 years had the highest 6-month mortality rates when compared with other age strata. Boumendil and colleagues found very old patients had comparably greater ICU and hospital-adjusted odds of death [16]. This has been similarly shown with the majority of deaths in older patients occurring either in the ICU or early following ICU discharge [9,15,20]. Whether these observed differences in short-term survival for very old patients are, in part, attributed to reduced treatment intensity, to the number of received interventions or to other factors remains uncertain. In our study, several nonmodifiable factors were predictive of hospital mortality including medical admission status, emergency surgery, primary neurologic, cardiac and gastrointestinal admission diagnoses, and admission from a chronic care facility.

Our study and prior available data, however, suggest that chronological age alone is probably insufficient to discriminate triage decisions on ICU admission. Rather, age probably represents an additive factor when coupled with frailty, physiologic reserve, burden of co-morbid illness, primary diagnosis, and illness severity. Prehospital disposition and/or functional status have been shown in numerous investigations to predict a worse clinical outcome [15,23,40]. This constellation of clinical factors probably has important bearing not only on short-term survival but also on long-term survival, neurocognitive performance, functional autonomy, and quality of life [6,12,15,20,29,41-43]. Accordingly, very old patients developing critical illness – who are characterized by a low burden of co-morbid disease, good function status, and no measurable frailty – are likely to benefit from ICU support. These relatively fit older patients may be characterized by a unique phenotype that portends greater physiologic reserve and resilience during episodes of critical illness [44,45]. We need more accurate and robust methods, however, for predicting quality-adjusted long-term survival and for optimizing therapeutic recovery in all patients ultimately admitted to the ICU – particularly very old patients, given their higher observed short-term mortality.

The present study has several limitations that should be considered. First, our study is prone to selection bias due to not capturing data on rates of ICU admission refusal and/or patient/surrogate preferences for not foregoing ICU support. Accordingly, we were not able to estimate the incremental gain of ICU support compared with ICU refusal in very old patients. Likewise, our study was not able to capture data on the process of triage, or the occurrence of bed shortages that may have prompted rationing of ICU beds.

Second, the APD does not collect data on additional variables that we would consider valuable, including cause of death, long-term (that is, 1-year) survival, health-related quality of life, neurocognitive outcomes, functional status, and measures of frailty. Our analysis is therefore largely descriptive and used the hospital discharge location (that is, rehabilitation/long-term care facility) as a surrogate. Third, we retrieved data only from centres that had consistently contributed to the APD during the study period; however, small variations in consistency and/or efficiency of data contribution may bias estimates and may impact generalizability across ANZ or other jurisdictions.

Fourth, we are unable to comment on additional clinical outcomes that are clearly important in this cohort, such as long-term functional status and cognitive decline. Finally, our estimated projections for resource demand are based on a number of assumptions that are prone to change (that is, annual changes in admission rate, median ICU/hospital lengths of stay, average cost per ICU patient-day, linear growth rate).

While crude, however, we contend that the data from this large cohort clearly identify the need for additional formal evaluations of ICU health resources to match the epidemiologic trends.

## Conclusions

In summary, the older population admitted to the ICU has grown rapidly. This growth is projected to continue and will have important implications on health resources in terms of triage, decision-making, expansion of ICU capacity, and advanced care planning. Very old patients in our study had lower short-term survival, which appeared to be influenced by prehospital function, co-morbid illness, surgical status, primary diagnosis, and illness severity. Survivors were also more likely to be transitioned to rehabilitation or long-term care facilities. We conclude that additional prospective investigations are urgently needed to better predict and improve the clinical outcomes for very old patients requiring ICU support and for preparation to match the expected demand on current ICU capacity.

## Key messages

• Very old patients represented 13.0% of all patients admitted to the ICU and increased by 5.6% annually during the study.

• Very old patients had lower short-term survival that was modified by prehospital function, co-morbid illness, surgical status, primary diagnosis, and illness severity.

• Survivors of critical illness were more likely to be transitioned to rehabilitation or long-term care facilities.

• These data imply a large projected need for ICU and hospital bed-days for very old patients within a decade.

## Abbreviations

ANZ: Australia and New Zealand; ANZICS CORE: Australian and New Zealand Intensive Care Society Clinical Outcomes and Resource Evaluation; APACHE: Acute Physiology and Chronic Health Evaluation; APD: Adult Patient Database; CI: confidence interval; ICU: intensive care unit; OR: odds ratio.

## Competing interests

The authors declare that they have no competing interests.

## Authors' contributions

SMB, SARW, AD, and RB participated in the conception and design of the study. CG, GKH, and DP contributed to data acquisition. SMB, SARW, AD, and RB performed and interpreted the data analysis. SMB, SARW, and RB drafted the manuscript. SMB, SARW, AD, CG, GKH, DP, and RB participated in critical revision of the manuscript. All authors read and approved the final manuscript.

## Supplementary Material

Additional data file 1 A Word file summarizing the operational definitions for pre-existing co-morbidities used in the study, based on the chronic health evaluation for APACHE II, APACHE III, and Simplified Acute Physiology Score II systems as outlined in the ANZICS APD data dictionary. Click here for file

Additional data file 2 A Word file containing a table that summarizes the characteristics and crude mortality of patients stratified by deciles of age strata. Click here for file

Additional data file 3 A Word file containing a table that summarizes the age-standardized sex-specific incidence rates of ICU admissions. Click here for file
