# Supplementary material for: Very old patients admitted to intensive care in Australia and New Zealand: a multi-centre cohort analysis
Source: Crit Care. 2009 Apr 1;13(2):R45. doi: 10.1186/cc7768 (PMC2689489; doi:10.1186/cc7768)
Supplement: Additional data file 2 — A Word file containing a table that summarizes the characteristics and crude mortality of patients stratified by deciles of age strata. [file cc7768-S2.doc]

**Additional Data File #2:** Characteristics and crude mortality of patients stratified by deciles of age strata.

| **Age Strata** | **18-29** | **30-39** | **40-40** | **50-59** | **60-69** | **70-79** | **>80** |
| --- | --- | --- | --- | --- | --- | --- | --- |
| **N (%)** | 8420 (7.0) | 8312 (6.9) | 12054 (10.0) | 18582 (15.5) | 25215 (21.0) | 31900 (26.6) | 15640 (13.0) |
| **Male sex (%)** | 58.3 | 55.7 | 57.5 | 62.0 | 64.2 | 60.4 | 51.1 |
| **Hospital Admission Source (%):**  **Home**  **Other Acute Care Hospital**  **Chronic Care Facility**  **Other ICU** | 75.1  21.5  0.6  2.7 | 73.7  23.1  0.6  2.5 | 75.8  20.9  0.8  2.4 | 79.9  17.6  0.8  1.7 | 81.0  16.5  0.9  1.6 | 81.0  16.3  1.3  1.5 | 79.8  15.6  3.3  1.3 |
| **Co-morbid disease (%):**  **Any**  **≥ 2** | 7.7  1.7 | 14.4  3.3 | 22.2  4.5 | 29.6  6.8 | 33.3  7.6 | 34.3  7.8 | 32.1  7.3 |
| **Specific co-morbid diseases (%):**  **Cardiovascular**  **Respiratory**  **Immunocompromised**  **Metastatic cancer**  **Hepatic**  **ESKD**  **Hematologic malignancy** | 1.5  2.4  3.0  0.6  1.0  1.0  1.1 | 3.1  3.9  4.6  1.4  3.6  2.1  1.4 | 6.2  5.1  5.5  2.6  5.8  2.8  1.8 | 12.3  7.8  6.5  3.9  4.0  3.1  2.2 | 18.0  10.1  5.6  3.7  2.1  3.6  2.1 | 21.9  10.7  4.5  3.1  0.9  4.1  1.7 | 23.5  9.5  3.2  2.3  0.5  4.1  1.1 |
| **Admission Details (%):**  **Non-elective admission**  **Surgical admission**  **Cardiovascular**  **Trauma**  **Emergency Surgical** | 14.4  29.4  10.3  31.7  68.6 | 18.8  30.1  16.7  18.0  56.4 | 28.6  38.7  30.9  11.0  39.7 | 41.7  50.4  45.3  5.9  26.4 | 47.6  56.7  54.1  3.6  23.8 | 46.7  56.6  54.1  3.5  25.9 | 38.2  53.0  39.5  5.6  38.0 |
| **Primary Diagnosis (%):**  **Sepsis/septic shock**  **Respiratory**  **Neurologic**  **Cardiac**  **Gastrointestinal (other)**  **Hepatic**  **Metabolic/poisoning**  **Gastrointestinal Bleeding** | 26.6  11.6  12.2  4.0  1.8  2.9  17.7  0.8 | 30.8  12.4  14.4  5.4  3.2  5.2  15.9  17.7 | 29.8  12.4  15.6  7.3  5.3  7.0  10.8  2.6 | 28.4  12.4  11.7  9.2  7.6  6.6  5.0  2.2 | 26.6  12.3  8.3  9.4  9.2  5.6  2.1  2.0 | 27.3  11.2  6.2  10.8  10.6  5.6  1.5  2.2 | 27.5  10.0  5.1  12.3  15.0  7.4  1.7  4.0 |
| **Illness severity scores:**  **APACHE II [mean (SD)]**  **non age-related APACHE II [mean (SD)]^¶^** | 12.8 (7.2)  12.8 (7.2) | 13.2 (7.6)  13.2 (7.6) | 14.5 (7.9)  13.4 (7.8) | 15.6 (7.6)  13.0 (7.6) | 17.1 (7.4)  13.0 (7.3) | 19.0 (7.2)  13.5 (7.2) | 19.8 (7.1)  13.8 (7.1) |
| **Mechanical Ventilation (%)** | 53.8 | 52.0 | 53.4 | 53.6 | 54.0 | 52.7 | 43.7 |
| **ICU Length of Stay [median (IQR)]** | 2.5 (1.6-5.2) | 2.5 (1.6-5.1) | 2.5 (1.6-5.5) | 2.4 (1.7-4.9) | 2.5 (1.7-4.8) | 2.7 (1.8-5.0) | 2.8 (1.8-4.9) |
| **Hospital Length of Stay [median (IQR)]** | 8.7 (4.2-19.2) | 9.1 (4.6-19.0) | 10.4 (5.8-21.0) | 10.8 (6.9-20.5) | 11.1 (7.2-20.8) | 12.8 (7.9-22.8) | 13.9 (8-24.7) |
| **ICU Mortality (%)** | 5.3 | 5.9 | 7.6 | 7.6 | 8.2 | 10.3 | 12.0 |
| **Hospital Mortality (%)** | 6.3 | 7.8 | 10.6 | 11.4 | 13.2 | 17.6 | 24.0 |
| **SMR (95% CI)** | 0.66 (0.49-0.84) | 0.83 (0.61-1.05) | 0.86 (0.68-1.03) | 1.02 (0.89-1.14) | 0.98 (0.9-1.07) | 1.02 (0.96-1.09) | 1.28 (1.19-1.36) |

Abbreviations: ESKD = end-stage kidney disease; SMR = standardized mortality ratio; ICU = intensive care unit; APACHE = acute physiology and chronic health evaluation
