# Supplementary material for: Very old patients admitted to intensive care in Australia and New Zealand: a multi-centre cohort analysis
Source: Crit Care. 2009 Apr 1;13(2):R45. doi: 10.1186/cc7768 (PMC2689489; doi:10.1186/cc7768)
Supplement: Additional data file 3 — A Word file containing a table that summarizes the age-standardized sex-specific incidence rates of ICU admissions. [file cc7768-S3.doc]

**Additional Data File #3:** Summary of age-standardized sex-specific incidence rates of ICU admission stratified by age strata compared with the sex-specific ratio from the 2001 Australian census.

| **Age Strata** | **Male Sex (%)** | | **Incidence Rate Ratio**  **(95% CI)** |
| --- | --- | --- | --- |
|  | **ICU Cohort** | **Australian Population^§^** |  |
| **18-29 (%)** | 4910 (58.3) | 1587858 (50.2) | 1.39 (1.33-1.45) |
| **30-39 (%)** | 4626 (55.7) | 1400432 (48.9) | 1.31 (1.26-1.37) |
| **40-49 (%)** | 6931 (57.5) | 1433273 (49.1) | 1.40 (1.35-1.45) |
| **50-59 (%)** | 11513 (62.0) | 1264436 (49.6) | 1.66 (1.61-1.71) |
| **60-69 (%)** | 16181 (64.2) | 854169 (49.8) | 1.81 (1.76-1.85) |
| **70-79 (%)** | 19281 (60.4) | 542351 (46.8) | 1.74 (1.70-1.78) |
| **≥80 (%)** | 7999 (51.1) | 270010 (37.1) | 1.77 (1.72-1.83) |
| **Overall** | 71441 (59.5) | 7352529 (48.7) | 1.55 (1.53-1.56) |

Abbreviations: ICU = intensive care unit

§ Data from the 2001 Australia census, Australia Bureau of Statistics [48].
